# Solid-state fermentation with *Rhizopus oligosporus* RT-3 enhanced the nutritional properties of soybeans

**DOI:** 10.3389/fnut.2022.972860

**Published:** 2022-09-08

**Authors:** Yongzhu Zhang, Ruicheng Wei, Fidelis Azi, Linshu Jiao, Heye Wang, Tao He, Xianjin Liu, Ran Wang, Baiyi Lu

**Affiliations:** ^1^Jiangsu Key Laboratory for Food Quality and Safety-State Key Laboratory Cultivation Base, Ministry of Science and Technology, Institute of Food Safety and Nutrition, Jiangsu Academy of Agricultural Sciences, Nanjing, China; ^2^Chemical Engineering Laboratory, Synthetic Biology and Intelligent Control Unit, Guangdong Technion Isreal Institute of Technology, Shantou, China; ^3^Key Laboratory for Quality Evaluation and Health Benefit of Agro-Products of Ministry of Agriculture and Rural Affairs, Key Laboratory for Quality and Safety Risk Assessment of Agro-Products Storage and Preservation of Ministry of Agriculture and Rural Affairs, College of Biosystems Engineering and Food Science, Zhejiang University, Hangzhou, China

**Keywords:** nutraceutical potential, enzyme, antioxidant activity, small peptide, soluble phenolic, metabolic pathway

## Abstract

Fermented soybean products are favorite foods worldwide because of their nutritional value and health effects. In this study, solid-state fermentation (SSF) of soybeans with *Rhizopus oligosporus* RT-3 was performed to investigate its nutraceutical potential. A rich enzyme system was released during SSF. Proteins were effectively transformed into small peptides and amino acids. The small peptide content increased by 13.64 times after SSF for 60 h. The antioxidant activity of soybeans was enhanced due to the release of phenolic compounds. The soluble phenolic content increased from 2.55 to 9.28 gallic acid equivalent (GAE) mg/g after SSF for 60 h and exhibited high correlations with microbial enzyme activities during SSF. The potential metabolic pathways being triggered during SSF indicated that the improved nutritional composition of soybean attributed to the biochemical reactions catalyzed by microbial enzymes. These findings demonstrated that SSF could evidently improve the nutritional value and prebiotic potential of soybeans.

## Introduction

Soybean has been cultivated for thousands of years in Asia, and its products are popular worldwide due to their abundant nutrition (1, 2). Proteins occupy approximately 40% of the dry weight (DW) of soybean making it an ideal material for various delicious protein foods with low cost compared with animal proteins (3). Soybean foods are considered promising substitutes of milk products for lactose-intolerant populations because of their free lactose property (4). The dietary consumption of soybean products shows health-promoting benefits, such as anti-diabetic, anticancer, anti-inflammatory, and anti-cardiovascular effects (1). At present, a wide range of soybean products have been made and the variety is still increasing.

Solid-state fermentation (SSF) with microorganism is an economically viable method used to biotransform food materials for nutritional enrichment, and serves a key role in forming new nutritional substances, including pigments, organic acids, and flavors (5). SSF with *Monascus anka* promotes the liberation of phenolics and enhances the antioxidant capacity of oats (6). SSF with *Saccharomyces cerevisiae* effectively improves the nutritional profiles of maize flour by decreasing antinutritional factors and increasing protein digestibility (7). In soybean food production, SSF is also used to produce microbial pigments, improve the levels of free amino acids, reduce the content of antinutritional factors, and enhance antioxidant ability (8). The content and composition of volatile compounds in tofu are significantly increased after fermentation with *Mucor* spp. (3). The co-SSF of soybean with *Actinomucor elegans* and *R. oligosporus* is effective in the hydrolysis of antinutritional factors and reduction of immunoreactivity (9). Tempeh is a traditional soybean food produced by SSF with strains of *Rhizopus* species, such as *R. oligosporus*, *Rhizopus arrhizus*, and *Rhizopus stolonifer*. During SSF, soybeans are covered with a thick white mycelium and the biomacromolecules are hydrolyzed into small-molecule nutrients with high bioavailability by fungal enzymes, thus improving the nutritional value and functional properties of soybeans (10). Thus far, studies on fermented soybean predominantly focus on flavor components, protein hydrolysis, and health-promoting effects, but little information is available on variations of microbial enzyme activities, their effects on small molecule nutrient production, and analysis of the potential biochemical reactions involved in the SSF of soybeans.

In this study, the variations of microbial enzyme activities, such as carbohydrate-hydrolyzing enzymes, esterase, and protease, derived from *R. oligosporus* RT-3 during SSF were studied. The protein degradation, small peptide production, phenolic content, and metabolite compositions were investigated. Afterward, the relationships among enzyme activities, small peptide content, phenolic content, and antioxidant activities were analyzed. Metabolites were finally tracked to their biosynthesis pathways to interpret the potential biochemical reactions during SSF.

## Materials and methods

### Materials and strain

The high purity (≥97%) standards, including ascorbic acid, vanillic acid, syringic acid, epicatechin, daidzin, glycitin, ferulic acid, genistin, daidzein, glycitein, quercetin, and genistein were purchased from Yuanye Bio-Technology Co., Ltd. (Shanghai, China). Other reagents of analytical grade. Soybeans (Liaodou, yellow seed coat, mainly planted in northern China, weigh 21.6 g of one hundred seeds) were obtained from a local market. *R. oligosporus* RT-3 isolated, screened and identified from Tempeh in our laboratory was used for soybean fermentation (11, 12). The strain was cultivated on potato dextrose agar at 30°C for 3 days. Then, spores produced were scraped in water (1 × 10^7^ spores/mL).

### Solid-state fermentation of soybeans

One kilogram soybeans were cleaned and then soaked in distilled water for 12 h. Subsequently, they were peeled and sterilized at 105°C for 0.5 h. After cooling to room temperature, 2 mL spore suspension (1 × 10^7^ spores/mL) was inoculated to 100 g soybeans. Soybeans were cultured in a constant temperature incubator at 30°C for 60 h. The fermented soybeans (FS) were sampled at 0, 12, 24, 36, 48, and 60 h. Wet samples were stored at −80°C for further study. The unfermented soybeans (US, 0 h) were used as control.

### Microbial enzymes

The enzymes released by *R. oligosporus* RT-3 during SSF were prepared in accordance with the report of Bei et al. (13). Briefly, 2 g wet sample was firstly ground using a mortar and then mixed with 10 mL citrate buffer (pH 5.5). The flasks were shaken at 30°C for 0.5 h to extract microbial enzymes. Subsequently, the mixture was centrifuged at 12,000 × *g* and 4°C for 0.5 h. The supernatant obtained was used as crude enzyme solution. The results of enzyme activities were expressed as per gram DW.

### α-Amylase and protease activities

α-Amylase and protease activities were measured in line with previous report (14). For α-amylase, 2 mL properly diluted enzyme solution was mixed with 1 mL acetate buffer (0.1 M, pH 4.5) and 1 mL of 1% (w/v) soluble starch at 37°C for 1 h. Then, the glucose content was determined using the 3,5-dinitrosalicylic acid (DNS) method. One unit of α-amylase was defined as the amount required to produce 1 μmol glucose per min. For protease, 1 mL enzyme solution was mixed with 1 mL casein solution (1%, w/v) with shaking at 40°C for 1 h and added with 10% trichloroacetic acid (2 mL) to stop the reaction. The mixture was centrifuged at 10,000 × *g* for 20 min. The absorbance was measured at 280 nm. One unit of protease was defined as the amount required to produce 1 μg tyrosine per min.

### β-Glucosidase and esterase activities

The determination of β-Glucosidase and esterase activities were concurred with previous report (15, 16). For β-glucosidase, 100 μL properly diluted sample was mixed with 900 μL citrate buffer (50 mM, pH 4.8) containing p-nitrobenzene-β-D glucoside (5 mM) at 50°C for 10 min. Two milliliters of 1.0 M Na_2_CO_3_ was added to stop the reaction. For esterase, 50 μL enzyme solution was mixed with 50 μL p-nitrophenyl acetate (150 mM) and 2.9 mL Tris-HCl buffer (9.2 mM, pH 7.5) at 25°C for 4 min. One unit of β-glucosidase and esterase was defined as the amount required to produce 1 μmol *p*-nitrophenol per min.

### Endoglucanase and exoglucanase activities

The endoglucanase and exoglucanase activities were measured according to the report of Ang et al. (17). For endoglucanase, 0.5 mL enzyme solution was mixed with 0.5 mL acetate buffer (0.05 M, pH 5.0) containing 2% (w/v) carboxymethylcellulose at 50°C for 0.5 h. For exoglucanase, 0.5 mL enzyme solution was mixed with 1 mL acetate buffer (0.05 M, pH 5.0) containing 50 mg WhatmanNo.1 filter paper at 50°C for 1 h. The glucose content was determined using the DNS method. One unit of endoglucanase and exoglucanase was defined as the amount required to produce 1 μmol glucose per min.

### Phytase activity

The phytase activity was measured based on the report of Acuña et al. (18). Briefly, 100 μL enzyme solution was mixed with 2.7 mL Tris-HCl (pH 7.0) containing 2.5 mM sodium phytate at 37°C for 0.5 h, and the mixture was added with 11.5 mL acetone–sulfuric acid–ammonium molybdate mixture (2:1:1). Eight milliliters of 1 M citric acid solution was used to stop the reaction. The mixture was centrifuged at 12,000 × *g* for 20 min. The absorbance of supernatant was read at 355 nm. One unit of phytase was defined as the amount required to produce 1 μmol inorganic phosphorus per min.

### Scanning electron microscope analysis

Soybeans were fixed in 2.5% (v/v) glutaraldehyde solution for 12 h. Then, soybeans were washed three times using distilled water and freeze-dried under −50°C. Afterward, soybeans were sputter-coated with platinum and then observed using SEM (EVO-LS10, Carl Zeiss AG, Germany).

### Soluble protein analysis

The soluble protein was obtained in accordance with the report of Huang et al. (9). The freeze-dried samples were ground into powder and prepared in Tris-HCl (50 mM, pH 8.2). The mixture was shaken at room temperature for 1 h and then centrifuged at 12,000 × *g* for 20 min. The protein content in the supernatant was determined using a bicinchoninic acid (BCA) assay kit. The supernatant was mixed with sample buffer solution and heated at 100°C for 5 min. Sodium dodecyl sulfate–polyacrylamide gel electrophoresis (SDS-PAGE) was performed using 4% stacking gel at 60 V for 0.5 h and 12% separating gel at 120 V for 1.5 h. The result was visualized using gel imaging systems (BIO-RADXR, Bio-Rad Laboratories, United States).

### Small peptide analysis

The small peptide content was analyzed in accordance with the report of Muhialdin et al. (19). The freeze-dried sample was prepared in water (1:10, w/v) and shaken for 1 h. After centrifugation at 12,000 × *g* for 20 min, the supernatant was filtered using ultrafiltration membrane (10 kDa, Millipore, United States). Subsequently, 100 μL filtrate was added to 7.5 mL reaction reagent for 2 min. The reaction reagent was composed of sodium tetraborate (25 mL, 50 mM), SDS (2.5 mL, 20%), o-phthaldialdehyde (40 mg), and β-mercaptoethanol (100 μL). The absorbance was read at 340 nm.

The peptide composition was analyzed using high-performance liquid chromatography (HPLC) equipped with the ZORBAX Eclipse Plus C18 reversed-phase analytical column (4.60 mm × 250 mm, 5 μm, Agilent) and diode array detector (G1315D) in accordance with the report of Rui et al. (20). Mobile phases were composed of water containing 0.1% trifluoroacetic acid (solvent A) and acetonitrile containing 0.1% trifluoroacetic acid (solvent B). Elution was carried out as follows: 0–20 min, 5–35% solvent B; 20–30 min, 35% solvent B. Twenty microliters sample was injected into the column. The flow rate and detection wavelength were 0.7 mL/min and 280 nm, respectively. The peptides in the sample were preliminarily analyzed according to retention time and peak area.

### Phenolic compound analysis

Soluble (free) phenolics (SPs) were extracted in accordance with the report of Bei et al. (13). Briefly, 6 g freeze-dried sample was added to 80% methanol. The mixture was subjected to ultrasonic treatment at 45°C for 1 h. After centrifugation at 12,000 × *g* for 20 min, the residue was treated with 80% methanol again. The combined supernatant was extracted with hexane to eliminate lipids followed by ethyl acetate extraction. The organic phase was concentrated with a rotary evaporator until dryness and then dissolved in 10 mL of 80% methanol for determination of the SP content (SPC).

Insoluble (bound) phenolics were commonly liberated by alkaline hydrolysis as previously described (13, 21, 22). The residue left after SP extraction was treated with 2 mol/L NaOH for 4 h. Subsequently, pH was regulated to 2.0. After centrifugation, the supernatant was extracted using the same method as the soluble fraction. The ethyl acetate fraction obtained was dissolved in 4 mL of 80% methanol for determination of the insoluble phenolic content (IPC). The phenolic content was measured using Folin-Ciocalteu method on the basis of the report of Xiao et al. (11).

The phenolic composition was analyzed using HPLC equipped with the ZORBAX Eclipse Plus C18 reversed-phase analytical column (4.60 mm × 250 mm, 5 μm, Agilent) and diode array detector (G1315D). The SP and IP fractions dissolved in 80% methanol above were filtered using a 0.22 μm syringe filter before HPLC analysis. The mobile phase was composed of water containing 0.4% acetic acid (solvent A) and acetonitrile (solvent B). Elution was carried out as follows: 9–75% solvent A for 0–40 min, 75–65% solvent A for 40–45 min, 65–50% solvent A for 45–50 min, and 50–95% solvent A for 50–55 min. Flow rate and detection wavelength were 0.8 mL/min and 280 nm, respectively. The phenolics in the sample were identified and quantified by their standards according to retention time and peak area.

### Antioxidant activity analysis

The IP and SP fractions at different fermentation time (0, 12, 24, 36, 48, and 60 h) were freeze-dried under −50°C and then dissolved in 80% methanol. The antioxidant activities of fermented soybean, including DPPH radical scavenging activity, ABTS radical cation scavenging activity, reducing power, and ferric reducing antioxidant power, were measured in accordance with the report of Yin et al. (23). For DPPH radical scavenging activity, 1 mL sample was mixed with 1 mL DPPH solution (0.2 mM) for 30 min and then the absorbance was read at 517 nm. For ABTS radical cation scavenging activity, ABTS.^+^ was prepared with the reaction of ABTS solution (7 mM) with K_2_S_2_O_8_ solution (2.45 mM). The mother liquor was diluted to 0.70 (OD_734 nm_). One milliliter sample was mixed with 4 mL ABTS.^+^ solution for 6 min and then the absorbance at 734 nm was read. For reducing power, 1 mL sample was mixed with 5 mL 0.2 M PBS (pH 6.6) and 5 mL 1% potassium ferricyanide at 50°C for 20 min. Trichloroacetic acid (5 mL, 10%, w/v) was added. Then, the mixture was centrifuged at 420 × *g* for 10 min. The supernatant (5 mL) was mixed with 1 mL ferric chloride (0.1%, w/v) for 10 min. The absorbance was measured at 700 nm. For ferric reducing antioxidant power, the reaction reagent was composed of 1 mL 10 mM TPTZ (in 40 mM hydrochloric acid), 1 mL 20 mM ferric chloride and 10 mL 0.3 M acetate buffer (pH 3.6). Two milliliters were mixed with 10 mL reaction reagent at 37°C for 20 min. The absorbance was measured at 593 nm.

### Metabolite profile analysis

The metabolite profile was analyzed in accordance with the report of Yin et al. (24). Briefly, 1 g freeze-dried sample was mixed with 5 mL reagent containing chloroform, methanol, and water (1:2.5:1, v/v/v) and supplemented with 40 μg/mL ribitol (internal standard). After centrifugation at 500 × *g* for 20 min, 2 mL supernatant was dried using a termovap sample concentrator and then supplemented with 50 μL of pyridine containing 20 mg/mL methoxyamine hydrochloride at 37°C for 60 min. Subsequently, 200 μL N-methyl-N-(trimethylsilyl)-trifluoroacetamide (MSTFA) containing 1% trimethylchlorosilane (TMCS) was added and the tube was kept under 70°C for 0.5 h. The supernatant obtained after centrifugation was analyzed using gas chromatography–mass spectrometry (GC-MS, TRACE 1300, flame ionization detector, 30 m × 250 μm × 0.25 μm elasticity quartz capillary, Thermo Fisher Scientific, United States). The operation conditions were as follows: stationary phase, hexane; purge flow rate of the helium carrier gas, 5 mL/min; inlet temperature, 250°C; MS transfer line temperature, 280°C; ion source temperature, 300°C; scan masses, m/z 40–600. The temperature programming of GC oven was shown as follows: 40°C, 0–1 min; 40–280°C, 1–40 min; and 280°C, 40–50 min. The metabolites (identity ≥ 80%) were analyzed by comparing the generated ion fragments in NIST mass spectral library (version 2.3, build 2017, prepared by NIST mass spectrometry data center). Five replicates were conducted.

### Statistical analysis

Data were expressed as an average of at least three replicates. One-way ANOVA followed by Duncan’s post–hoc multiple comparisons were performed in SPSS. *p* < 0.05 indicated significance. Also, bivariate correlations were performed to obtain the Pearson correlation coefficient (PCC) in SPSS.

## Results

### Trends of enzyme activities during solid-state fermentation

**FIGURE 1:**
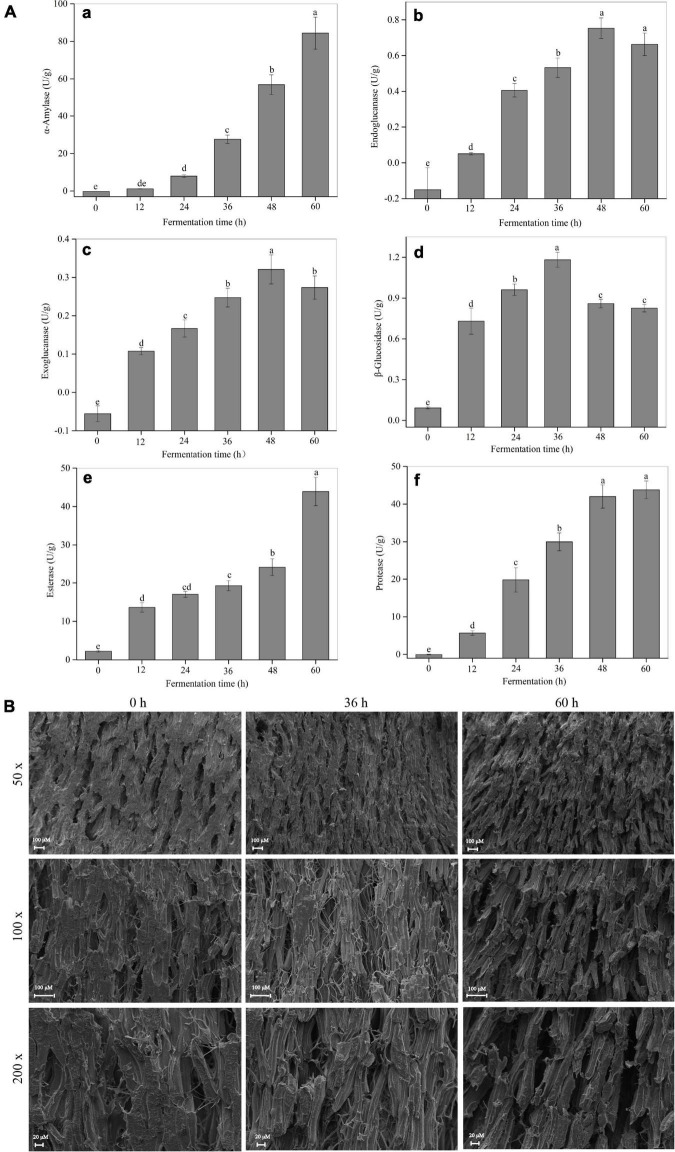
Changes of microbial enzyme activities and microstructure observation during SSF. **(A)** a, α-amylase; b, endoglucanase; c, exoglucanase; d, β-glucosidase; e, esterase; f, protease. **(B)** Microstructure. *p* < 0.05 was considered as the significant level. The small letters were used to show the significant difference between samples at different fermentation time.

As shown in Supplementary Figure 1, the filamentous fungi *R. oligosporus* RT-3 grew well on soybeans accompanied by the formation of a large number of white hyphae during SSF. An abundant enzyme system released by microorganisms is the material basis for their growth and proliferation (25, 26). Therefore, the changing trends of several key enzymes released by *R. oligosporus* RT-3 were analyzed. As shown in Figure 1A, a rapid increase of the α-amylase activity was observed during the whole SSF. The enzyme activities of cellulases, including endoglucanase and exoglucanase, increased and peaked at 48 h and then suffered a slight reduction. The β-glucosidase exhibited a trend similar to cellulase, which increased before 36 h and then slightly decreased. Similar to α-amylase, esterase and protease increased continuously during the whole fermentation. Additionally, SEM analysis indicated that relatively large voids were produced after SSF, which might be due to the degradation of biomacromolecules induced by fungal enzymes released from *R. oligosporus* RT-3 (Figure 1B).

### Effects of solid-state fermentation on soluble proteins and small peptides

**FIGURE 2:**
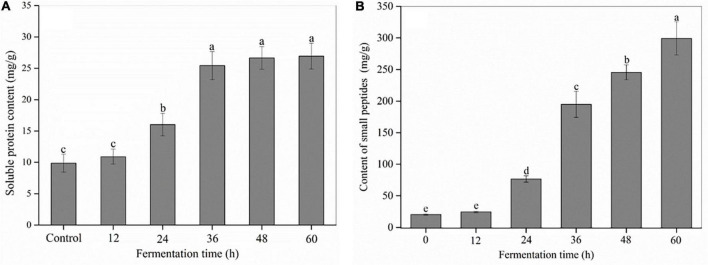
The content of soluble proteins and small peptides in soybeans during the SSF. **(A)** Soluble proteins; **(B)** small peptides. *p* < 0.05 was considered as the significant level. The small letters were used to show the significant difference between samples at different fermentation time.

As shown in Figure 2A and Supplementary Figure 2, SSF with *R. oligosporus* RT-3 made a significant difference on the content and composition of soluble proteins in soybeans. The soluble protein content exhibited an evident increase with the extension of fermentation time. In the control (0 h), several bands, such as α′ and β subunits of β-conglycinin, and acidic and basic subunits of glycinin were observed at molecular mass ranging from 20 to 150 kDa (9). However, the protein bands except some small proteins with a molecular mass less than 20 kDa almost completely disappeared after SSF for 36 h, indicating that macromolecular proteins were degraded into small soluble proteins, peptides or amino acids during SSF.

Small peptides refer to peptides with molecular weight less than 10 kDa (27). As shown in Figure 2B, *R. oligosporus* RT-3 apparently increased the total content of small peptides in soybeans during SSF. For example, the content of small peptides at 60 h was 14.64 times of that in the control. The increase in the total content of small peptides was due to the protease released by *R. oligosporus* RT-3 during SSF, which was further confirmed by the high PCC (*R*^2^ = 0.974, *p* < 0.01) between protease and the total content of small peptides. HPLC showed that approximately 90 small peptide peaks were found in US and FS (Supplementary Figures 3A–F). The areas of 11 major peaks at retention time ranging from 3 to 20 min were significantly changed during SSF. The fractions eluted at early retention times were considered peptides with low molecular weight and high hydrophilicity (20). For *R. oligosporus* RT-3 fermentation, most high peaks were presented at retention times before 10 min. Furthermore, the production of relatively large and increased hydrophobic peptides (peaks 10 and 11) was observed.

### Trends of phenolic content and composition during solid-state fermentation

As shown in Supplementary Figure 4, SPC showed a slight increase from 0 to 24 h, a rapid increase from 24 to 36 h, and then a slight increase again from 36 to 60 h. For example, SPC reached 9.28 mg gallic acid equivalent (GAE)/g in FS at 60 h, which was 3.64 times of control. IPC increased from 0 to 24 h and then significantly decreased from 24 to 60 h. Furthermore, HPLC indicated that SSF with *R. oligosporus* RT-3 changed the phenolic composition of soybeans (Supplementary Figure 5 and Table 1). For SPs, the contents of vanillic and ferulic acids increased first and then decreased during SSF. The contents of epicatechin, daidzein, glycitein, and genistein exhibited a continuous increase during SSF. However, continuous decreases in the contents of syringic acid, daidzin, glycitin, and genistin were observed. For insoluble phenolics (IPs), the contents of vanillic acid, syringic acid, daidzin, ferulic acid, genistin, glycitein, and quercetin increased first and then decreased during SSF. The contents of epicatechin, glycitin, and genistein showed continuous increases. However, daidzein showed a continuous decrease during SSF. Besides, soy isoflavones were effectively transformed into glycosides after SSF. Notably, the insoluble forms of syringic, vanillic, and ferulic acids, respectively, occupied 82.57, 78.77, and 71.04% of their total contents (soluble + insoluble forms) in US, and this finding was in line with the report of Adom and Liu (28). The relationships between enzyme activities and phenolic contents were further analyzed. As shown in Supplementary Table 1, enzyme activities, i.e., α-amylase (*R*^2^ = 0.938, *p* < 0.01), showed high correlations with SPC. A similar result was also observed on the content of the total phenolics (SPC + IPC), demonstrating that the enzymes released by *R. oligosporus* RT-3 during SSF significantly improved the liberation of phenolic compounds.

**TABLE 1:** Contents of the phenolic compounds in soybeans during SSF.

Phenolic components (ug/g)		Fermentation time (h)					
		0	12	24	36	48	60
Vanillic acid	SP	5.50 ± 0.37^De^	6.81 ± 0.49^Df^	25.26 ± 1.21^Cef^	131.29 ± 8.99^Ad^	34.03 ± 1.55^Bf^	27.98 ± 1.84^BCf^
	IP	20.41 ± 1.05^Cd^	22.83 ± 1.23^Cd^	33.51 ± 3.81^Ad^	27.07 ± 1.98^Bc^	22.59 ± 1.48^Ce^	16.09 ± 1.73^De^
Syringic acid	SP	17.65 ± 1.51^Ae^	17.17 ± 1.09^Aef^	14.26 ± 1.12^Bf^	9.80 ± 0.81^Ch^	4.44 ± 0.47^Dg^	N.D.
	IP	83.60 ± 5.36^Cb^	89.62 ± 6.81^BCb^	128.32 ± 12.26^Ab^	101.29 ± 7.31^Bb^	94.25 ± 10.33^BCb^	79.56 ± 5.27^Ca^
Epicatechin	SP	14.50 ± 0.81^Ce^	18.06 ± 1.46^Bef^	18.26 ± 1.56^Bf^	20.52 ± 2.71^ABh^	21.02 ± 1.74^ABfg^	23.03 ± 2.51^Afg^
	IP	12.06 ± 1.02^Ce^	13.19 ± 0.84B^Cde^	22.17 ± 1.77^Be^	25.51 ± 1.78^Bc^	32.69 ± 2.84^Ad^	32.97 ± 2.83^Ac^
Daidzin	SP	263.87 ± 32.10^Ab^	256.53 ± 7.95^Ab^	246.07 ± 18.79^Ab^	104.51 ± 8.14^Be^	94.92 ± 7.66^Bd^	77.37 ± 9.61^Be^
	IP	4.31 ± 0.25^CDf^	4.81 ± 0.36^Cef^	14.03 ± 0.61^Aef^	6.00 ± 0.63^Bd^	4.57 ± 0.46^Cfg^	3.64 ± 0.25^Dfg^
Glycitin	SP	87.76 ± 5.56^Ac^	86.30 ± 10.08^ABc^	74.73 ± 6.66^BCd^	73.30 ± 6.86^Cf^	69.41 ± 4.94^CDe^	58.70 ± 4.39^De^
	IP	0.78 ± 0.04^Df^	0.80 ± 0.03^Df^	1.06 ± 0.14^Cg^	1.54 ± 0.17^Bd^	2.77 ± 0.21^Afg^	2.99 ± 0.15^Ag^
Ferulic acid	SP	50.93 ± 3.48^Bd^	61.53 ± 4.78^Ad^	54.07 ± 6.22^Bde^	26.31 ± 3.00^Ch^	14.11 ± 0.97^Dfg^	N.D.
	IP	124.95 ± 11.57^Ba^	136.55 ± 18.46^Ba^	206.69 ± 15.42^Aa^	219.44 ± 18.40^Aa^	119.66 ± 10.59^Ba^	64.90 ± 4.79^Cb^
Genistin	SP	675.23 ± 31.31^Aa^	670.90 ± 36.47^Aa^	626.13 ± 51.48^Aa^	446.19 ± 29.36^Ba^	350.14 ± 30.46^Ca^	345.07 ± 25.51^Cb^
	IP	50.01 ± 3.09^Dc^	52.16 ± 4.37^Dc^	84.08 ± 7.47^Bc^	110.57 ± 8.21^Ab^	62.26 ± 5.22^Cc^	23.98 ± 2.57^Ed^
Daidzein	SP	78.74 ± 4.38^Ec^	78.19 ± 6.99^Ecd^	134.48 ± 12.98^Dc^	201.87 ± 16.18^Cb^	262.27 ± 11.87^Bb^	370.77 ± 24.58^Aa^
	IP	1.28 ± 0.09^Af^	1.01 ± 0.12^Bf^	0.93 ± 0.07^BCg^	0.91 ± 0.11^BCd^	0.76 ± 0.03^Cg^	N.D.
Glycitein	SP	30.41 ± 2.19^Cde^	33.61 ± 1.96^Ce^	33.46 ± 2.26^Cef^	49.11 ± 6.19^Bg^	58.71 ± 5.92^Be^	120.80 ± 10.07^Ad^
	IP	1.00 ± 0.05^Bf^	1.06 ± 0.03^Bf^	1.42 ± 0.07^Ag^	N.D.	N.D.	N.D.
Quercetin	SP	11.90 ± 0.76^Ae^	11.69 ± 0.52^Aef^	12.16 ± 0.91^Af^	11.47 ± 1.01^Ah^	11.69 ± 0.93^Afg^	11.87 ± 0.75^Afg^
	IP	2.07 ± 0.26^Bf^	2.44 ± 0.19^Bef^	3.12 ± 0.20^Afg^	2.39 ± 0.25^Bd^	2.26 ± 0.17^Bfg^	2.32 ± 0.19^Bg^
Genistein	SP	82.26 ± 4.97^Ec^	98.13 ± 8.22^DEc^	121.47 ± 13.15^Dc^	160.08 ± 10.47^Cc^	207.78 ± 30.61^Bc^	252.80 ± 19.03^Ac^
	IP	1.33 ± 0.10^Ef^	1.69 ± 0.19^Ef^	4.32 ± 0.47^Dfg^	5.20 ± 0.22^Cd^	10.93 ± 0.61^Af^	7.75 ± 0.81^Bf^

*p* < 0.05 was considered as the significant level. The capital letters were used to show the significant difference between different samples for the same component. The small letters indicated the significant difference between the different components within the same sample. N.D., not detected.

### Trends of antioxidant activities of soybeans during solid-state fermentation

**FIGURE 3:**
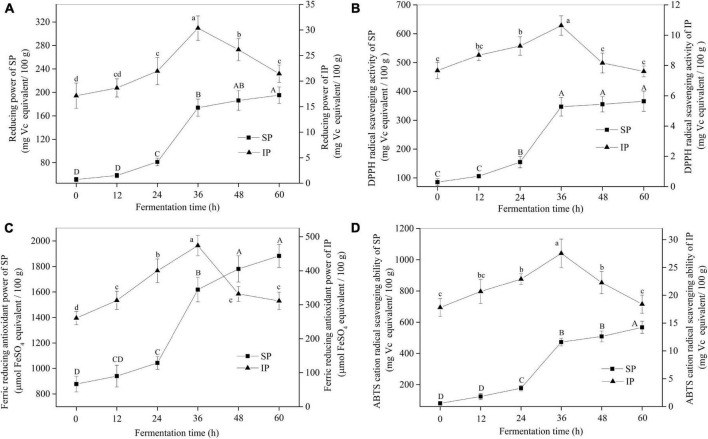
The antioxidant activities of soybeans during SSF. **(A)** Reducing power; **(B)** DPPH radical scavenging activity; **(C)** ferric reducing antioxidant power; **(D)** ABTS cation radical scavenging ability. *p* < 0.05 was considered as the significant level. The small letters were used to show the significant difference between samples at different fermentation time for IP. The capital letters indicated the significant difference between samples at different fermentation time for SP. SP, soluble phenolics; IP, insoluble phenolics.

As shown in Figure 3A, the reducing power of soybeans for SPs evidently increased during SSF. For example, the reducing power of soybeans at 60 h increased by 2.81 times of US. The trends of antioxidant activity of soybeans in other assays showed a similar result with the reducing power for SP fraction (Figures 3B–D). Additionally, the antioxidant activity of SP was much higher than that of IP. However, the antioxidant activity for IP fraction in four antioxidant assays increased first, peaked at 36 h, and then decreased rapidly. Furthermore, as shown in Supplementary Table 2, high correlations (e.g., *R*^2^ = 0.991 for reducing power, *p* < 0.01) were observed between antioxidant activities and SPC, indicating that the antioxidant activities of soybean were predominantly derived from the high content of phenolic compounds especially soluble fractions.

### Metabolite profile analysis

**FIGURE 4:**
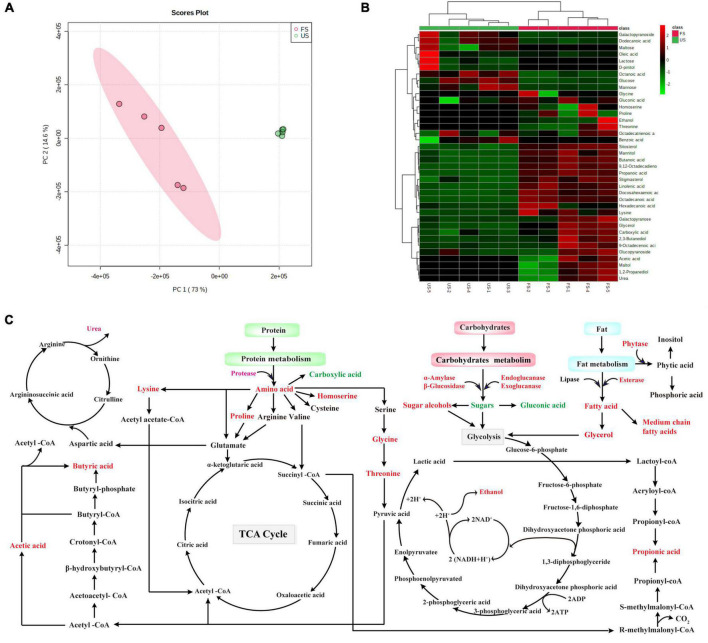
The analysis of metabolite profiles in US and FS and the potential metabolic pathways involved during SSF. **(A)** PCA biplot; **(B)** heatmap dendrogram; **(C)** metabolic pathways involved during SSF. Upregulated metabolites: red color; Downregulated metabolites, green color; Undetected metabolites, black color. US, unfermented soybean; FS, fermented soybean.

The metabolite profiles of US and FS at 36 h were analyzed using GC-MS (Table 2). A total of 38 kinds of metabolites were identified in soybeans. The principal component analysis (PCA) was used to analyze the changes in metabolite profiles. As shown in Figure 4A, the principal components PC1 and PC2 contributed to 73 and 14.6% of the total variance in metabolite compositions, respectively. For PC1, a significant difference was observed between US and FS, demonstrating that the nutritional compositions of soybeans were apparently altered by *R. oligosporus* RT-3 fermentation. For the small component PC2, results indicated that the nutritional composition of FS only had a slight similarity with that of US. The results of PCA biplot were further confirmed by the analysis of a cluster dendrogram (Figure 4B).

**TABLE 2:** Metabolites detected by GC-MS analysis in US and FS.

Metabolites	Area % of each sample		Metabolites	Area % of each sample	
	US	FS		US	FS
**Carbohydrates**			Octanoic acid	12.19 ± 5.45^a^	0.16 ± 0.15^b^
Glucose	8.38 ± 5.67^a^	0.12 ± 0.07^b^	Oleic acid	0.43 ± 0.23^a^	0.05 ± 0.02^b^
Galactopyranose	0.90 ± 0.33^a^	1.06 ± 0.71^a^	9-Octadecenoic acid	0.53 ± 0.61^a^	1.10 ± 0.90^a^
Galactopyranoside	14.81 ± 6.64^a^	0.54 ± 0.23^b^	9,12-Octadecadienoic acid	3.27 ± 0.67^b^	51.82 ± 3.04^a^
Glucopyranoside	1.074 ± 1.509^a^	0.26 ± 0.22^a^	Total fatty acids	27.38 ± 8.16^b^	64.76 ± 10.19^a^
Lactose	16.73 ± 8.51	N.D.	**Amino acids**		
Maltose	1.47 ± 0.95	N.D.	Glycine	N.D.	0.26 ± 0.09
Mannose	0.86 ± 0.68^a^	0.01 ± 0.00^b^	Homoserine	N.D.	0.39 ± 0.27
Total carbohydrates	44.21 ± 5.57^a^	1.99 ± 1.18^b^	Lysine	0.50 ± 0.34^a^	0.48 ± 0.32^a^
**Alcohols**			Proline	N.D.	0.38 ± 0.36
D-pinitol	7.03 ± 7.03	N.D.	Threonine	N.D.	0.10 ± 0.04
Ethanol	N.D.	0.45 ± 0.17	Total amino acids	0.50 ± 0.34^b^	1.60 ± 0.66^a^
Glycerol	2.38 ± 1.16^b^	6.48 ± 2.07^a^	**Organic acids**		
Maltol	N.D.	0.24 ± 0.07	Acetic acid	N.D.	4.18 ± 1.35
Mannitol	0.12 ± 0.03^a^	0.07 ± 0.01^b^	Benzoic acid	0.62 ± 0.37	N.D.
1,2-Propanediol	N.D.	0.32 ± 0.20	Butanoic acid	1.46 ± 0.75^b^	3.46 ± 0.15^a^
2,3-Butanediol	7.53 ± 5.78^a^	10.75 ± 5.77^a^	Carboxylic acid	1.65 ± 1.12^a^	0.89 ± 0.25^a^
Total alcohols	17.05 ± 7.87^a^	18.30 ± 7.85^a^	Gluconic acid	0.11 ± 0.05^a^	0.02 ± 0.00^b^
**Fatty acids**			Propanoic acid	0.11 ± 0.05^b^	2.65 ± 0.29^a^
Docosahexaenoic acid	0.55 ± 0.33^a^	0.65 ± 0.23^a^	Total organic acids	3.95 ± 0.67^b^	11.20 ± 1.51^a^
Dodecanoic acid	0.64 ± 0.27^a^	0.04 ± 0.01^b^	**Others**		
Hexadecanoic acid	6.38 ± 2.32^a^	8.04 ± 7.23^a^	Stigmasterol	1.96 ± 1.11^a^	0.44 ± 0.09^b^
Linolenic acid	0.07 ± 0.02^a^	0.09 ± 0.03^a^	Sitosterol	4.94 ± 2.31^a^	1.22 ± 0.15^b^
Octadecatrienoic acid	3.23 ± 2.81^a^	0.44 ± 0.15^a^	Urea	N.D.	0.50 ± 0.43
Octadecanoic acid	0.11 ± 0.05^b^	2.36 ± 0.84^a^			

*p* < 0.05 was considered as the significant level. The small letters indicated the significant difference for the same component between US and FS. N.D., not detected.

Metabolites could be classified into parent categories (Table 2), which were composed of carbohydrates, alcohols, organic acids, fatty acids, amino acids, and a miscellaneous group. Most of carbohydrates, including monosaccharides (glucose and mannose) and disaccharides (lactose and maltose) and their derivatives (galactopyranoside), apparently decreased after SSF. The total level of carbohydrates significantly decreased from 44.21 to 1.99% after fermentation. The decreases in carbohydrate levels indicated that they were used as energy and carbon sources for the growth of *R. oligosporus* RT-3 during SSF. Additionally, some carbohydrates might be converted into alcohols, and this phenomenon was further verified by the increased levels of maltol, glycerol, ethanol, and 1,2-propanediol. The level of mannitol in FS suffered a decrease after fermentation. Several fatty acids were identified in US and FS. The total level of fatty acids, including saturated and unsaturated medium-chain fatty acids, increased by 1.37 times that of US after SSF. Prominent increases in the contents of octadecanoic and 9,12-octadecadienoic acids were obtained after SSF. The level of 9,12-octadecadienoic acid in FS significantly increased by 14.85 times that of US. The levels of oleic and octanoic acids suffered decreases after SSF. Only 1 kind of free amino acids was detected in US. However, the species of amino acids increased from 1 to 5 in FS after fermentation. All levels of free amino acids except lysine in soybeans were significantly increased after SSF, indicating that *R. oligosporus* RT-3 fermentation significantly improved the release of amino acids in soybeans, which was in line with the trend of small peptides above. SSF with *R. oligosporus* RT-3 also significantly increased the total levels of organic acids in soybeans. The levels of short-chain fatty acids (SCFAs), including acetic, propanoic, and butanoic acids, exhibited apparent increases after SSF, which might be due to the biotransformation of dietary fiber in soybeans by *R. oligosporus* RT-3 (29). The level of gluconic acid evidently decreased after SSF with *R. oligosporus* RT-3. However, the level of benzoic acid significantly (*p* < 0.05) decreased, suggesting that it was degraded by *R. oligosporus* RT-3 during SSF (30). Furthermore, SSF with *R. oligosporus* RT-3 increased the level of urea and decreased the levels of stigmasterol and β-sitosterol.

## Discussion

Soybeans and their products have been a crucial component of our daily diet due to their abundant nutritional value, such as high contents of proteins and unsaturated fatty acids (2, 3). Fermented soybean-based foods are popular worldwide because of their delicious taste and health-promoting effects. SSF with edible fungi is considered as an economically biological enzymatic hydrolysis process that is commonly used in the soybean industry. In the present study, a rich enzyme system composed of α-amylase, esterase, cellulase, protease, and β-glucosidase was secreted into soybeans by *R. oligosporus* RT-3 during SSF. These enzymes could hydrolyze various biological macromolecules, such as proteins, fats, and carbohydrates, into small-molecule nutrients with relatively high bioavailability and produce secondary metabolites with functional properties (5). This hydrolysis of microbial enzymes was also confirmed by the changes in soybean microstructure during SSF, wherein an increasingly loose spatial structure was observed by SEM.

Proteins occupy approximately 40% of the DW of soybeans, making soybeans as an ideal protein source for human. SSF with *R. oligosporus* RT-3 significantly affected their contents and compositions. During SSF, the macromolecular proteins in soybean were effectively hydrolyzed by the protease released from *R. oligosporus* RT-3. Soybean is listed as one of the “big eight” food allergens that can lead to allergic reaction in about 5–8% of children and 2% of adults (31). Glycinin (including acidic and basic subunits) and β-conglycinin (including α′, α, and β subunits), major allergenic proteins (9), were highly degraded in the present study. This phenomenon suggested that the immunoreactivity of soybeans might be reduced by SSF with *R. oligosporus* RT-3. The gradual increase in the content of small peptides indicated that most soybean proteins were degraded into small peptides and free amino acids during SSF, and this finding was further confirmed by the correlation analysis between protease activities and small peptide content. However, the HPLC analysis at 280 nm in this study could only determine the peptides containing aromatic amino acids, which limited the integrity of the composition analysis of small peptides. In the next step, we will use other methods to further analyze the information of small peptides. Reports have proved that the small peptides generated by *in vitro* enzymatic hydrolysis and fermentation are beneficially associated with a multitude of metabolic activities, such as reducing blood pressure and cholesterol, inhibiting of the bile acid reabsorption in the intestinal tract and platelet aggregation as well as anticancer, antioxidative, antimicrobial, and immunoregulatory activities (1, 32). In addition, the content of free essential amino acids, which cannot be synthesized in human body and served as important component in cell, significantly increased after fermentation (Table 2). The increased contents of small peptides and essential amino acids induced by SSF with *R. oligosporus* RT-3 might improve the prebiotic potential of soybeans. Therefore, SSF with *R. oligosporus* RT-3 can be a feasible strategy to increase the bioavailability of soybean proteins and health-promoting potential.

Phenolic compounds have been paid considerable attention because of their health-promoting effects, which predominantly exist in insoluble forms, and are covalently bound to the structural components of cell wall, such as cellulose, hemicellulose, lignin, pectin, and proteins (33). The absorption of bound phenolics in the gastrointestinal tract is dependent on the release of sugar moiety. In this work, we found that SSF with *R. oligosporus* RT-3 effectively improved the release of phenolic compounds in soybeans by increasing the content of SPs. As reported by Bei et al. (13), SSF with *M. anka* evidently enhanced the phenolic content of oat powder especially the ferulic and vanillic acids. Razak et al. (34) also found that SSF with *R. oligosporus* and *M. purpureus* apparently improved the release of phenolic compounds, such as ferulic, sinapic, vanillic, caffeic, syringic, and 4-hydroxybenzoic acids, in rice bran. Interestingly, the IPC increased before 36 h and then decreased, which was possibly because the strong covalent bonds formed by soybean components, like protein and cellulose, with insoluble phenolic compounds were weakened by the hydrolytic enzymes derived from *R. oligosporus* RT-3 during the early stage of SSF, making their extraction easier than those in the control, and later liberated or degraded (35). Additionally, isoflavones, including glycoside (daidzin, genistin, and glycitin) and aglycone (daidzein, genistein, and glycitein) forms, are the major phenolic compounds found in soybeans. Isoflavones predominantly occur as glycoside in US. Reports showed that unfermented soybean foods commonly have a higher level of glycoside isoflavones than aglycones. The former is considered to exhibit a lower bioactivity than the latter because the aglycone form is more easily absorbed by the gastrointestinal tract. Thus, the potential bioactivities of isoflavones are suppressed by their conjugated glycosides (36). However, during SSF with *R. oligosporus* RT-3, glycoside isoflavones were effectively biotransformed into their corresponding aglycones. As reported by Zheng et al. (37), enzymes, such as cellulase and amylase, secreted by microorganisms during fermentation are demonstrated to destroy the cell walls of cereals and transform the IPs into free form, thus improving the release of phenolic compounds. The PPC between SPC and enzyme activities during SSF indicated that the enzymes secreted by *R. oligosporus* RT-3 were deeply involved in the liberation of phenolic compounds. The carboxylic groups in the phenolic compound structure can link with biomacromolecules, such as sugar and proteins, by ester bonds (33), which can be broken by carbohydrate-hydrolyzing enzymes, and esterase released by *R. oligosporus* RT-3 during SSF. Furthermore, the destruction of the original structure of soybeans by enzymatic hydrolysis made the extraction of phenolic compounds easy. Therefore, the increase in the phenolic content of soybeans was predominantly due to the rich enzyme system secreted by *R. oligosporus* RT-3. Reports have shown that soybean polyphenols especially isoflavones are important phenolic sources in our daily diet, which are beneficial to human health by preventing several diseases, including cerebral ischemia, cancer, and hyperlipidemia and regarding chronic diseases, such as myocardial infarction, insulin resistance, stroke, and systemic inflammation (1, 38). Hence, the increased content of available phenolics induced by SSF could further improve the prebiotic potential of soybeans. The most outstanding bioactivity of phenolic compounds is the antioxidant activity for the aromatic phenolic ring in their structure, which displays free radical-scavenging capacity (25). Different antioxidant compounds in foods may work against oxides through different mechanisms (39). Hence, four assays, including DPPH radical scavenging activity, ABTS radical cation scavenging activity, reducing power, and ferric reducing antioxidant power were used to evaluate the antioxidant activities of soybeans in the present study. The antioxidant activities of soybeans significantly increased during SSF and high correlations were obtained between antioxidant activities and phenolic contents, indicating that the antioxidant activity of soybeans was predominantly derived from their high content of phenolic compounds. Previous studies have also reported that SSF with filamentous fungi evidently improves the phenolic content and the antioxidant ability of scavenging free radical (6, 8, 34). Additionally, the phenolic extracts were not purified in this study. Hence, other compounds like antioxidant peptides might also contribute to the antioxidant capacity of soybeans as previous report (40). Free radical can oxidize proteins, lipids, and DNA; affect mitochondrial function; activate caspase-3; destroy mitochondrial function; and promote apoptosis, consequently causing several diseases such as neurodegenerative disease (23). The fermented soybean with higher free radical scavenging activity could effectively attenuate oxidative stress in our body, thus exhibiting health-promoting potential.

Although many SSF-based studies are conducted on soybeans and their products, a gap still exists in understanding the biochemical pathways involved in SSF. The metabolomic analysis of US and FS were further performed using GC-MS. Figure 4 exhibited the links within these pathways, which were possibly triggered during the SSF of soybeans. Long-chain polymeric carbohydrates were degraded into small-molecule sugars by enzymes, including α-amylase, β-glucosidase, endoglucanase, and exoglucanase, derived from *R. oligosporus* RT-3 and finally entered the glycolysis pathway and tricarboxylic acid cycle (TCA) or biotransformed into sugar alcohols to provide energy and carbon source for the growth of *R. oligosporus* RT-3 during SSF. The increase in ethanol could apparently improve the aroma of soybeans. Also, the dietary fiber conversion by *R. oligosporus* RT-3 resulted in the synthesis of three major SCFAs, acetic, propionic, and butyric acids. As reported by Koh et al. (41), acetic acid may be derived from pyruvate via acetyl-CoA or via the Wood-Ljungdahl pathway. Propionic acid can be produced from phosphoenolpyruvate via the succinate or the acrylate pathway, wherein lactic acid is converted into propionic acid. Butyrate can be formed from two molecules of acetyl-CoA followed by the synthesis of acetoacetyl-CoA, β-hydroxybutyryl-CoA, crotonyl-CoA, butyryl-CoA, and butyryl-phosphate. SSF with *R. oligosporus* RT-3 significantly increased the contents of SCFAs, including acetic, propionic, and butyric acids. As reported by Mirzaei et al. (42), SCFAs are generated as waste product for microorganisms and are necessary for balancing redox state in anaerobic conditions. However, SCFAs display health-promoting effects on host cellular activities, including differentiation, immune modulation, inflammation, and energy metabolism. Hence, the significant increase in the contents of SCFAs after SSF could improve the bioactive potential of soybeans. Long-chain lipids were hydrolyzed into saturated and unsaturated medium-chain fatty acids by the lipases derived from *R. oligosporus* RT-3. According to a consistency of bulk experimental data and epidemiologic studies, the dietary consumption of essential polyunsaturated fatty acids can decrease the risk for urolithiasis, chronic inflammation, and urinary tract tumor (43). Some free fatty acids can serve as flavors or flavor precursors to enhance the quality of soybeans (44). Proteins were hydrolyzed into their constituent small peptides and amino acids by proteases derived from *R. oligosporus* RT-3, which could assist the absorption of amino acids in the gut and attenuate bitterness by the hydrolysis of soy proteins from the C- or N-terminus. Also, some amino acids might be synthesized by glycolysis or transamination, degraded via a range of catalyzing reactions, and entered the TCA or urea cycle. Phytic acid, one of the major antinutritional factors in soybean-based foods, is originally synthesized to store phosphorus in plant seeds. Metal cations, such as Mg^2+^, Ca^2+^, Zn^2+^, and Fe^2+^, can be attracted and chelated by the negative charge produced by phytic acid, thereby forming insoluble phytates, which make it hard for monogastric animals to absorb divalent cations (45). In the present study, a high level of phytase activity released from *R. oligosporus* RT-3 was observed during SSF, which indicated that phytic acid in soybeans might be hydrolyzed during SSF (Supplementary Figure 6). As described above, macromolecular components in soybean were effectively degraded into small molecular nutrients that are easily absorbed, and some active substances, such as essential amino acids, small peptides, phenolic compounds, and SCFAs, were released or produced during SSF with *R. oligosporus* RT-3, indicating that SSF with *R. oligosporus* RT-3 significantly improved the nutrient compositions and bioactive potentials of soybeans.

## Conclusion

This work revealed that *R. oligosporus* RT-3 secreted a rich enzyme system, such as protease, esterase and carbohydrate hydrolase, into soybeans during SSF. The biomacromolecules, such as proteins, fats and carbohydrates in soybeans, were hydrolyzed into small-molecule nutrients, such as small peptides, free amino acids, medium-chain fatty acids, and simple sugars, which were easier to be digested in human gastrointestinal tract. IPs were effectively transformed into SPs by SSF with *R. oligosporus* RT-3, thus increasing the antioxidant activities of soybeans. Soy isoflavones were also transformed into glycosides after SSF. The dietary fiber conversion by *R. oligosporus* RT-3 resulted in the synthesis of three major SCFAs, acetic, propionic, and butyric acids. The potential metabolic pathways being triggered during SSF demonstrated that the improved nutrient profile and prebiotic potential of soybeans attributed to the biochemical reactions catalyzed by microbial enzymes. Our findings provided a strong support for the add value of SSF on soybeans.

## Data availability statement

The raw data supporting the conclusions of this article will be made available by the authors, without undue reservation.

## Author contributions

RW and BL: resources, conceptualization, data curation, funding acquisition, and writing-review and editing. YZ: investigation, methodology, formal analysis, writing-original draft, and visualization. RCW, FA, LJ, HW, TH, and XL: methodology and writing-review and editing. All authors contributed to the article and approved the submitted version.

## Abbreviations

ABTS: 2,2-azinobis(3-ethylbenzothiazoline-6-sulfonic acid) diammonium salt
BCA: bicinchoninic acid
DNS: 3,5-dinitrosalicylic acid
DPPH: 2,2-diphenyl-1-picrylhydrazyl
DW: dry weight
FS: fermented soybean
GAE: gallic acid equivalent
GC-MS: gas chromatography-mass spectrometry
HPLC: high-performance liquid chromatography
IPs: insoluble phenolics
IPC: insoluble phenolic content
MSTFA: N-methyl -N-(trimethylsilyl)-trifluoroacetamide
PCC: Pearson correlation coefficient
PCA: principal component analysis
SCFAs: short-chain fatty acids
SDS-PAGE: sodium dodecyl sulfate-polyacrylamide gel electrophoresis
SEM: scanning electron microscope
SPs: soluble phenolics
SPC: soluble phenolic content
SSF: solid-state fermentation
TCA: tricarboxylic acid cycle
TMCS: trimethylchlorosilane
TPTZ: 2,4,6-tris(2-pyridyl)-*s*-triazine
US: unfermented soybean
Vc: ascorbic acid.
