# Supplementary material for: Solid-state fermentation with Rhizopus oligosporus RT-3 enhanced the nutritional properties of soybeans
Source: Front Nutr. 2022 Sep 8;9:972860. doi: 10.3389/fnut.2022.972860 (PMC9493129; doi:10.3389/fnut.2022.972860)
Supplement: Supplementary file 1 [file Data_Sheet_1.docx]

**Figure legends**

**Supplementary Figure 1** The appearance and morphology of soybeans during SSF

**Supplementary Figure 2** Effects of SSF on the composition of soluble proteins

**Supplementary Figure 3** HPLC analysis of the compositions of small peptides (< 10 kDa) during SSF

**Supplementary Figure 4** The phenolic content of soybeans during SSF. IPC, insoluble phenolic content; SPC, soluble phenolic content.

**Supplementary Figure 5** HPLC chromatograms of phenolic composition in soybeans during SSF. (A) A mixture of 11 standards; (B) SPs in US; (C) SPs in FS; (D) IPs in US; (E) IPs in FS. 1, vanillic acid; 2, syringic acid; 3, epicatechin; 4, daidzin; 5, glycitin; 6, ferulic acid, 7, genistin; 8, daidzein; 9, glycitein; 10, quercetin; 11, genistein. SPs, soluble phenolics; IPs, insoluble phenolics; US, unfermented soybean; FS, fermented soybean.

**Supplementary Figure 6** The phytase activity in soybeans during SSF


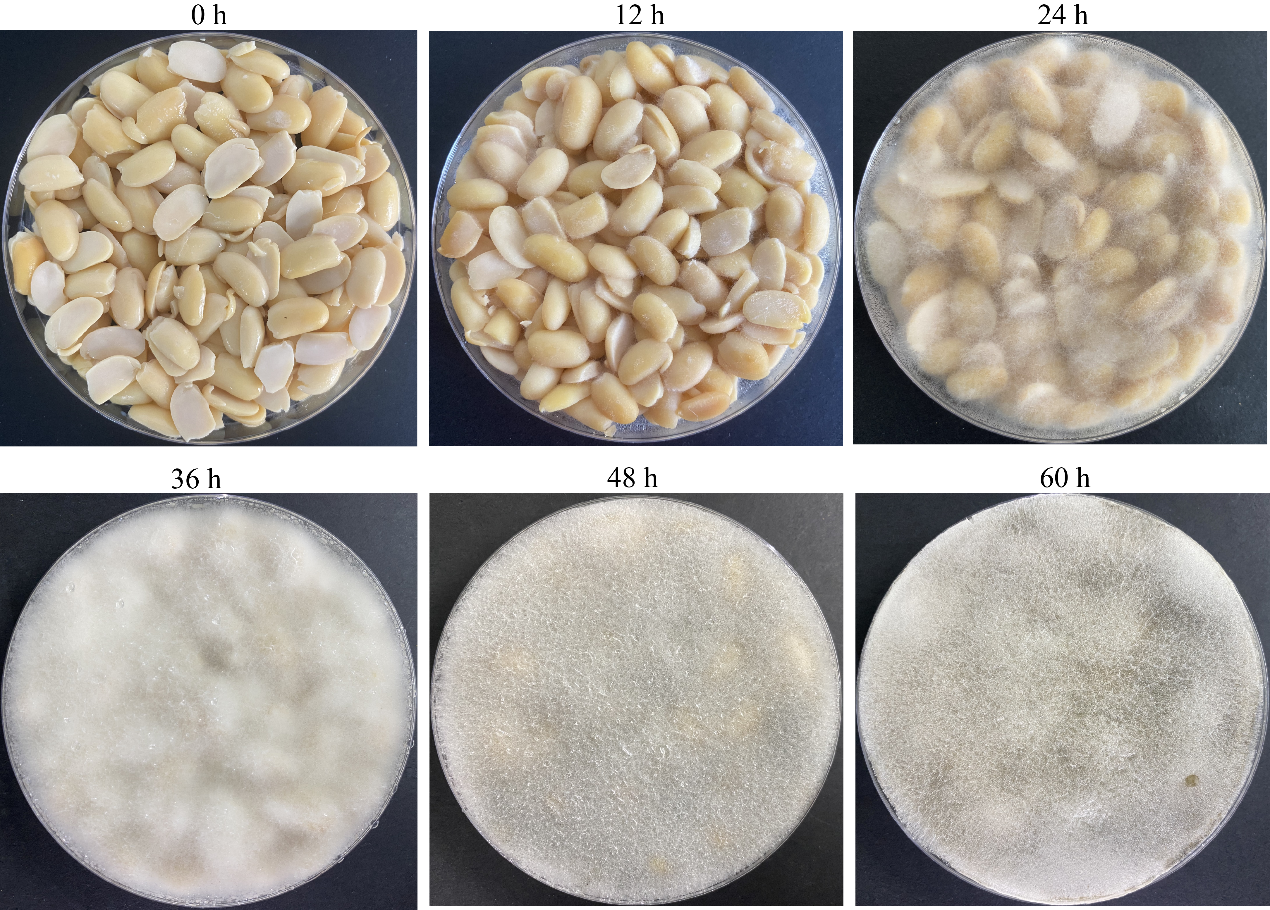


**Supplementary Figure 1**

**
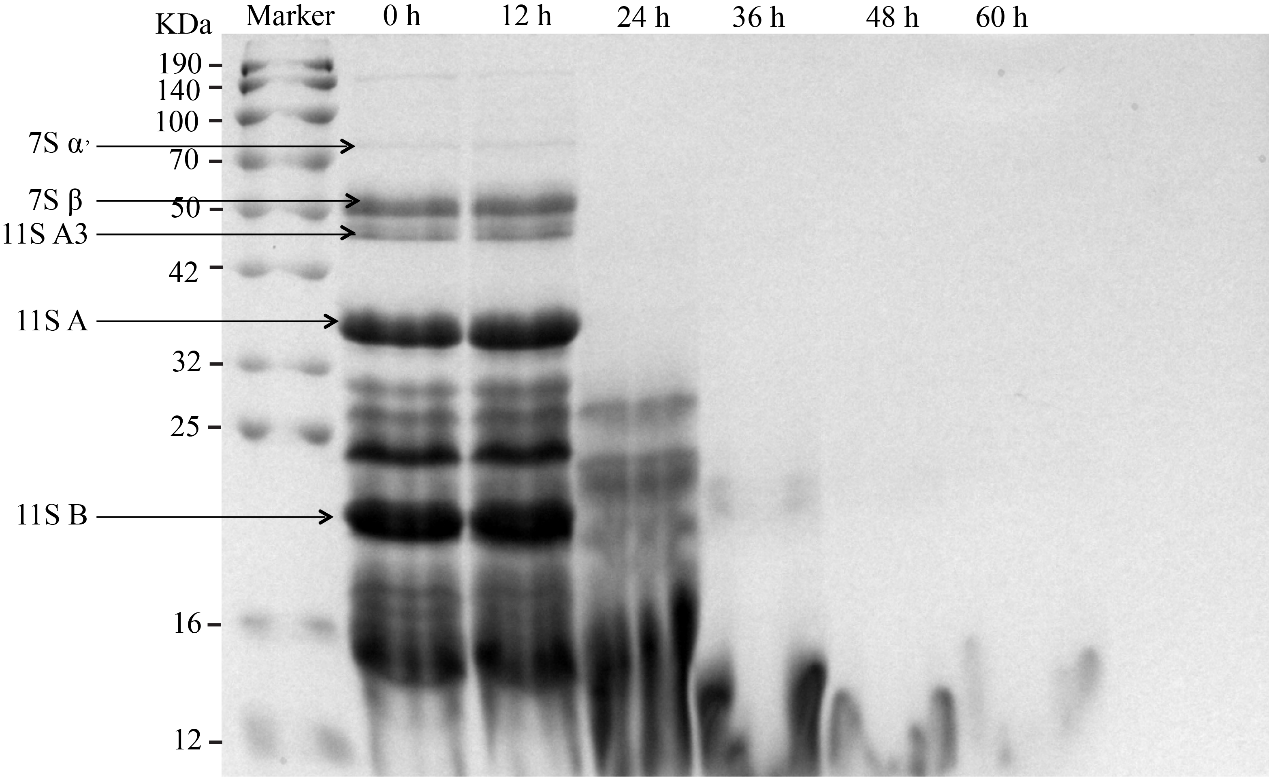
**

**Supplementary Figure 2**

**
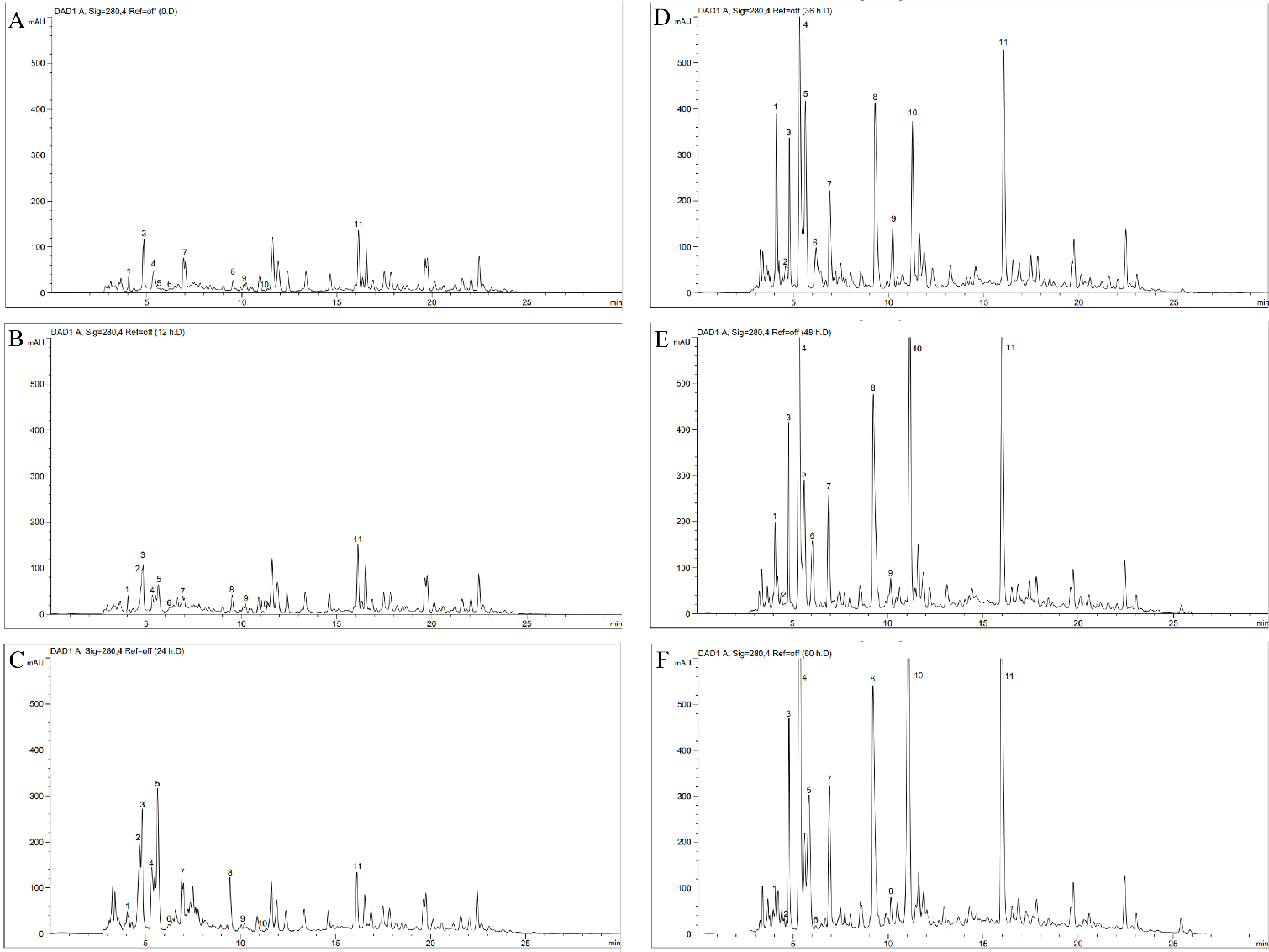
**

**Supplementary Figure 3**

**

**

**Supplementary Figure 4**

**
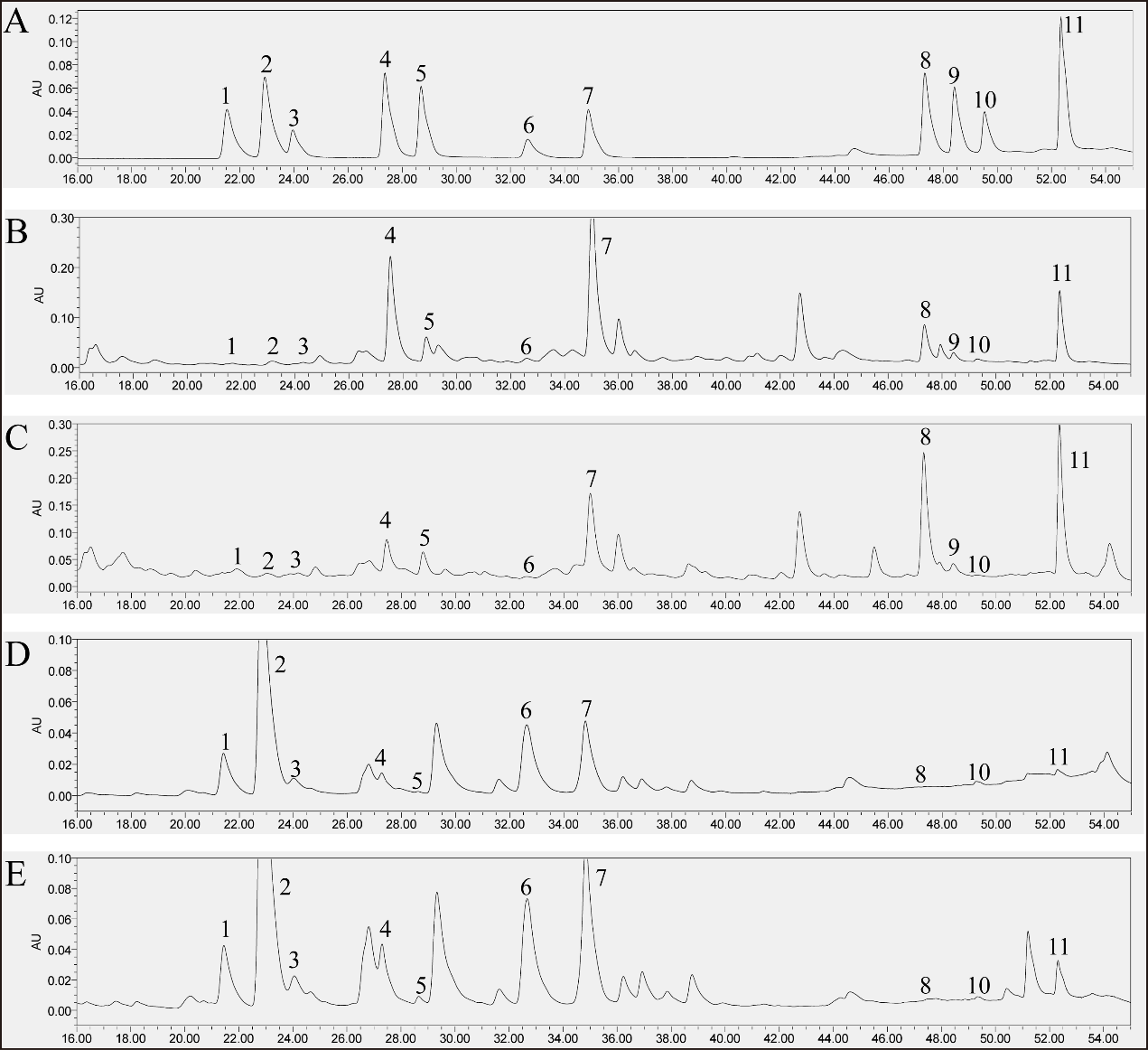
**

**Supplementary Figure 5**

**

**

**Supplementary Figure 6**

**Supplementary Table 1** The PCC between enzyme activities and phenolic contents during SSF

| composition | α-Amylase | Endoglucanase | Exoglucanase | β-Glucosidase | Esterase | Protease |
| --- | --- | --- | --- | --- | --- | --- |
| SPC | 0.938^**^ | 0.873^*^ | 0.844^*^ | 0.505 | 0.837^*^ | 0.945^**^ |
| SPC+IPC | 0.927^**^ | 0.888^*^ | 0.861^*^ | 0.539 | 0.832^*^ | 0.952^**^ |

Significant difference was represented as asterisks: ^∗∗^ 0.001 < *p* < 0.01, ^∗^ 0.01 < *p* < 0.05.

**Supplementary Table 2** The PCC between antioxidant activities and phenolic contents during SSF

| Composition | Reduce power | DPPH radical scavenging activity | Ferric reducing antioxidant power | ABTS radical cation scavenging activity |
| --- | --- | --- | --- | --- |
| SPC | 0.991^**^ | 0.982^**^ | 0.997^**^ | 0.994^**^ |
| IPC | -0.239 | 0.423 | 0.205 | 0.243 |

Significant difference was represented as asterisks: ^∗∗^ 0.001 < *p*< 0.01, ^∗^ 0.01 < *p* < 0.05.
